# Interaction of Newly Platinum(II) with Tris(2-carboxyethyl)phosphine Complex with DNA and Model Lipid Membrane

**DOI:** 10.1007/s00232-017-9972-z

**Published:** 2017-07-25

**Authors:** Hanna Pruchnik, Teresa Kral, Martin Hof

**Affiliations:** 1 Department of Physics and Biophysics, Wrocław University of Environmental and Life Sciences, ul. Norwida 25, 50-375 Wrocław, Poland; 2 J. Heyrovsky Institute of Physical Chemistry of the ASCR, v.v.i., Dolejškova 2155/3, 182 23 Prague 8, Czech Republic

**Keywords:** Platinum(II) complex, DNA, DPPC bilayer, TCSPC-FCS, IR spectroscopy, DSC

## Abstract

Structural properties of plasmid DNA and model lipid membrane treated with newly synthesized platinum(II) complex *cis*-[PtCl_2_{P(CH_2_CH_2_COOH)_3_}_2_] (*cis*-DTCEP for short) were studied and compared with effects of anticancer drug cisplatin, *cis*-[Pt(NH_3_)_2_Cl_2_] (*cis*-DDP for short). Time Correlated Single Photon Counting Fluorescence Correlation Spectroscopy (TCSPC-FCS) was employed to study interactions between those platinum complexes and DNA. The TCSPC-FCS results suggest that bonding of *cis*-DTCEP derivative to DNA leads to plasmid strain realignment towards much more compact structure than in the case of *cis*-DDP. Application of both differential scanning calorimetry and infrared spectroscopy to platinum complexes/DPPC showed that *cis*-DTCEP slightly increases the phospholipid’s main phase transition temperature resulting in decreased fluidity of the model membrane. The newly investigated compound—similarly to *cis*-DDP—interacts mainly with the DPPC head group however not only by the means of electrostatic forces: this compound probably enters into hydrophilic region of the lipid bilayer and forms hydrogen bonds with COO groups of glycerol and PO_2_^−^ group of DPPC.

## Introduction

Platinum compounds are very effective in the treatment of ovarian and testicular cancers and in combinations with other antitumor agents are used in the therapy of many carcinomas, e.g., leukemia bladder, breast, small lung, head and neck tumors (Brabec and Kasparkova 2005; Wheate et al. 2010; Dasari and Tchounwou 2014; Florea and Büsselberg 2011; Alama et al. 2009). Cisplatin, *cis*-DDP, (*cis*-[PtCl_2_(NH_3_)_2_]) is one of the most potent anticancer agents, however its application is limited mainly due to the acquired resistance to cisplatin and severe toxic effects in normal tissues, including nephrotoxicity, neurotoxicity, ototoxicity (Dasari and Tchounwou 2014; Hou et al. 2009).

The aim of synthesis of new analogs of platinum is to increase effectiveness and selectivity against cancer tissues and most of all—decrease the toxicity against health cells. Differences in both activity and toxicity of new complexes of tin stem from the presence of different ligands. The type of the ligand can influence the number and type of DNA adducts and also has significant influence on pharmacokinetics because only the uncombined fraction of the medicine can have a pharmaceutical effect (Brabec and Kasparkova 2005; Florea and Büsselberg 2011; Alama et al. 2009; Pivonková et al. 2006; Pizarro and Sadler 2009; Onoa et al. 1998; Alam et al. 2014). Recently, a new platinum-based compound containing a phosphine ligand tris(2-carboxyethyl)phosphine [P(CH_2_CH_2_COOH)_3_] (TCEP) instead of ammonium group and two chlorine atoms in *cis* and *trans* conformation with potential anticancer activity has been introduced. The phosphane complexes exhibit cytotoxic activity against cisplatin resistant tumor cells and sometimes are more active than cisplatin (Pruchnik et al. 2015; Henklewska et al. 2017). Newly obtained platinum(II) complex with tris(2-carboxyethyl)phosphine (TCEP): *cis*-[PtCl_2_(TCEP)_2_], which is the subject of this research exhibits interesting reactivity in solutions (Pruchnik et al. 2015). Our preliminary studies show that *cis*-DTCEP is a very promising antitumor agent against GL-1 (leukemia B), CL-1 (lymphoma T), CLBL-1 (lymphoma B) and breast tumors CMT-U309, CMT-U27. Cytotoxic activity of the new platinum complex(II) was much higher than that of cisplatin and the cell death was associated with apoptosis. Interestingly, the tested compound differently affected the cell cycle progression than cisplatin (Henklewska et al. 2017).

Cisplatin and other platinum-containing drugs are believed to induce apoptosis in cancer cells by covalently binding to DNA; however, they also react with cell membranes, peptides, and proteins (Dasari and Tchounwou 2014; Hou et al. 2009; Pivonková et al. 2006; Pizarro and Sadler 2009; Onoa et al. 1998; Alam et al. 2014; Peleg-Shulman et al. 2001; Wiglusz and Trynda-Lemiesz 2014; Pruchnik et al. 2015; Oberoi et al. 2013). Moreover, it is believed that the severe side effects such as neurotoxicity and cellular resistance are related to the interplay of cisplatin with biological membranes (Dasari and Tchounwou 2014; Florea and Büsselberg 2011). Furthermore, drug-membrane interaction plays an important role in drug transport, distribution, and accumulation. The most possible explanation of *cis*-DTCEP toxicity seems to be its ability to interact with DNA and lipid membrane. Thus, having investigated properties of this compound in our earlier work (Pruchnik et al. 2015), this time our research was focused on examining the interaction between new platinum(II) complex and biomolecules, such as plasmid DNA, 1,2-dipalmitoyl-sn-glycero-3-phosphocholine (DPPC) and comparing obtained results with the results for well-known cisplatin. For this we used spectroscopy and calorimetrical methods. In particular the effect of platinum complexes on plasmid DNA was investigated using single-molecule fluorescence technique, time correlated single photon counting fluorescence correlation spectroscopy (TCSPC-FCS). Interaction between *cis*-DTCEP and model lipid membranes were studied by the means of differential scanning calorimetry (DSC) and Fourier transform infrared spectroscopy (FTIR).

## Materials and Methods

### Chemicals

The platinum(II) complex with tris(2-carboxyethyl)phosphine (*cis*-[PtCl_2_{P(CH_2_CH_2_COOH)_3_}_2_], *cis*-DTCEP for short, was prepared by procedures reported earlier (Pruchnik et al. 2015). The 1,2-dipalmitoyl-sn-glycero-3-phosphatidylcholine (DPPC) was purchased from Sigma Aldrich (Poznań, Poland).

### Differential Scanning Calorimetry (DSC)

Samples for DSC were prepared from multilamellar liposomes (MLV) of phosphatidylcholine (DPPC) according to the procedure described previously (Pruchnik et al. 2014). MLVs prepared from pure lecithin (control sample) and lecithin with *cis*-DTCEP were placed in Mettler Toledo standard aluminum crucibles of 40 μl capacity. Tightly closed vessels were incubated for two days at 4 °C. The measurements were performed with Mettler Toledo Thermal Analysis System D.S.C. 821^e^, operated at the heating rate of 2 °C/min from 20 to 60 °C. Thermal cycles were repeated three times. The data were analyzed using original software provided by Mettler Toledo.

### Attenuated Total Reflectance Fourier Transform Infrared Spectroscopy (ATR-FTIR)

The IR method was applied as described earlier (Pruchnik 2017) with a few modifications. DPPC dissolved in chloroform was placed on ZnSe crystal and dried under nitrogen for a few minutes and under vacuum for 24 h. The dried films of DPPC were hydrated for 4 h in aqueous solution of *cis*-DTCEP above the main phase transition of DPPC. Molar ratio of DPPC/*cis*-DTCEP was 0.2. Measurements were performed using Thermo Nicolet 6700 MCT spectrometer (Thermo Fisher Scientific, Waltham, MA) with ZnSe crystal at a heating cycle from 20 to 50 °C. Each single spectrum was obtained from 128 records at 2 cm^−1^ resolution in the range of 700–4000 cm^−1^. Preliminary elaboration of the spectrum was done using the EZ OMNIC v 8.0 program, also by Thermo Nicolet. After filtering the noise out of the extract spectrum, the spectrum of the water solution was removed and the baseline corrected.

### Time Correlated Single Photon Counting Fluorescence Correlation Spectroscopy (TCSPC-FCS)

TCSPC-FCS measurements were performed on the MicroTime 200 inverted confocal microscope (PicoQuant, Germany). The pH βApr-1-Neo plasmid (10 kbp and contour length 3.4 μm) was a generous gift from the laboratory of Prof. Maciej Ugorski (Department of Biochemistry and Molecular Biology, Wrocław University of Environmental and Life Sciences Wroclaw, Poland). An appropriate amount of the DNA plasmid stock solution prepared in deionized nuclease-free water (Sigma Aldrich) labeled with PicoGreen^®^ (Molecular Probes) (C_PicoGreen_/C_DNA base pair_ ratio of 0.02) was accordingly added both to the *cis*-DDP and *cis*-DTCEP solutions and incubated for 24 h (Kral et al. 2005). All samples were measured at 25 °C.

## Results and Discussion

Broadly performed research of cisplatin allowed to get some insight into mechanisms of its activity. Among other things its mode of action has been linked to its ability to cross link with the purine bases on the DNA, interfering with DNA repair mechanisms, causing DNA damage, and subsequently inducing apoptosis in cancer cells. Several additional mechanisms, other than DNA-platination, have also been indicated as the source of the cytoxicity of platinum drugs. For example platinum complexes can react with a number of non-DNA cellular components such as glutathione. Cisplatin is prone to interact with phosphatidylserine and other phospholipid components of the cellular membranes and thus modulate their function (Oberoi et al. 2013; Dasari and Tchounwou 2014).

Relating to those publicized results we started investigating our newly synthesized compound (*cis*-DTCEP) from fundamental research. In our previous work we investigated the interaction of the compound with glutathione (Pruchnik et al. 2015) and in this research we focused on investigating molecular interactions between *cis*-DTCEP and phospholipids and DNA, which (as noted earlier) was the first step before starting the research on the cellular level.

### DNA Binding Experiments

**Fig. 1:**
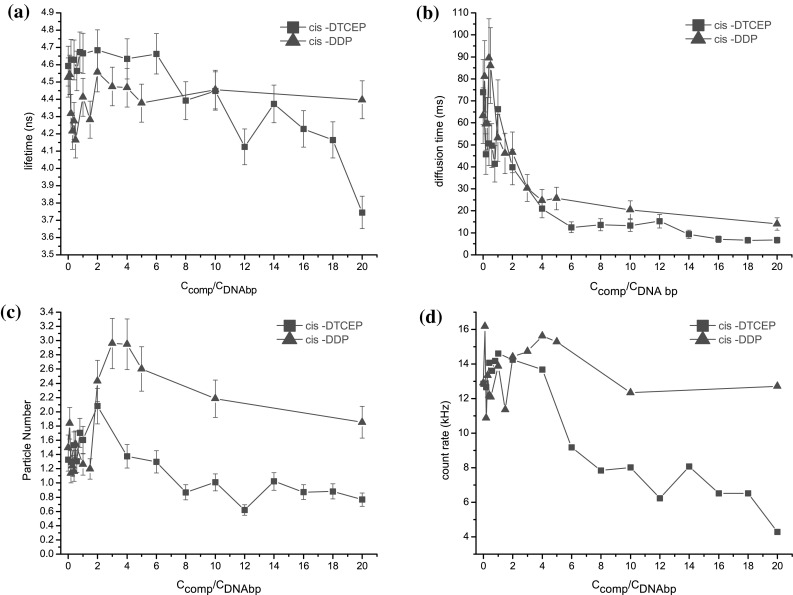
Variation of the lifetime (**a**) diffusion time, (**b**) particle number, (**c**) and count rate (**d**) values as a function of C_compound_/C_DNA bp_ ratio. C_PicoGreen_/C_DNA bp_ = 0.02

To identify whether the activity is related to interaction with DNA molecules, DNA binding properties of cisplatin complexes were investigated. Experiments were performed over a wide range of *cis*-DDP and *cis*-DTCEP concentrations, expressed here as C_comp_/C_DNA bp_ ratio, varying from 0 up to 20 molecules of the compound per one DNA base pair. Figure 1a, b, c, and d show the lifetime, the diffusion time, the particle number, and the count rate dependence on C_comp_/C_DNA bp_. Additionally, based on the fact that PicoGreen^®^ exhibits changes in fluorescence lifetime when bounded to folded and unfolded regions of the DNA molecule, TCSPC-FCS allows for a detailed insight into the supramolecular quality folding (Adjimatera et al. 2006). Plotting of the mean fluorescence lifetime versus C_compound_/C_DNAbp_ < 1 showed no significant changes for *cis*-DTCEP and a slight decrease from 4.6 to 4.1 ns for *cis*-DDP/DNA complex. Interestingly, estimated diffusion time of the plasmid decreased from 75 ms in water to 45 ms in the presence of *cis*-DTCEP but increased to 90 ms in the presence of *cis*-DDP. Particle number (PN) and count rate (CR) values experienced fluctuation but on average remained unchanged. Further titration of the plasmid with *cis*-DDP revealed an increase of the lifetime (from 4.1 to 4.5 ns), the particle number (from 1.2 to 3.0) and the count rate (from 12 to 15 kHz) all peaked at 4 molecules per one DNA base pair. Simultaneously, more than threefold decrease (from 90 to 25 ms) of the diffusion time was observed. Growth of both the particle number and the count rate is not self-explanatory. The C_compound_/C_DNA bp_ dependence of PN and CR is due to the apparent increase in fluorophore concentration as a result of multiple labeled DNA strain being adducted in the presence of the examined complexes (Adjimatera et al. 2006; Pruchnik et al. 2016). In contrary, an increasing number of the *cis*-DTCEP molecules per DNA base pair (C_comp_/C_DNA bp_ ratios from 1.0 to 4) yielded no significant change in lifetime and count rate. The particle number increased, reaching maximum for 2 *cis*-DTCEP molecules per base pair then decreased down to 1.3 (Fig. 1c). Simultaneously a 2-fold decrease (from 40 to 20 ms) of the diffusion time was observed as an evidence of persistent change of the DNA conformation.

The behavior of the diffusion time value change is not difficult to understand: at the beginning there are quite a lot of binding sites on DNA for platinum(II) complexes, thus the number of bound molecules of the complex increases with time resulting in continuous variation of the DNA structure. When binding sites are becoming saturated with *cis*-DDP or *cis*-DTCEP plasmid DNA structure changes are slowed down and finally stabilized. We were able to follow stabilization of supramolecular assemblies within the C_comp_/C_DNA bp_ range from 4 to 20. As plasmid DNA was interacting with an increasing amount of *cis*-DTCEP compound, a significant decrease (from 4.7 to 3.7 ns) of the lifetime was observed. Simultaneously there was no lifetime change observed for *cis*-DDP/DNA assembly. The diffusion time decrease (from 25 to 15 ms and from 20 to 5 ms) together with the particle number (from 3.0 to 2.0 and from 1.4 to 0.8) and the count rate (from 15 to 12 kHz and from 14 to 4 kHz) was observed for *cis*-DDP and *cis*-DTCEP, respectively. The lifetime drop refers to the changed microenvironment of PicoGreen^®^ (Dragan et al. 2010). Compacted DNA molecules start to precipitate, which probably causes further particle number and count rate decrease. Therefore significantly higher decrease in the lifetime, the diffusion time, the particle number and the count rate indicated that *cis*-DTCEP interaction caused more of global change in DNA conformation (Humpolícková et al. 2008), which is not the case for *cis*-DDP complex.

In summary, there is a resemblance between *cis*-DTCEP and *cis*-DDP: both modify DNA structure. However, the newly synthesized platinum complex *cis*-DTCEP which leads to a tightly packed supramolecular assembly is much more effective than *cis*-DDP.

### Interactions between DPPC and cis-DTCEP

Interactions of *cis*-DTCEP and *cis*-DDP with dipalmitoylphosphatidylcholine bilayer were investigated using differential scanning calorimetry and additionally—with the help of infrared spectroscopy. More accurately speaking, the effect of these complexes on the phase transitions and fluidity of the DPPC was examined.

**Fig. 2:**
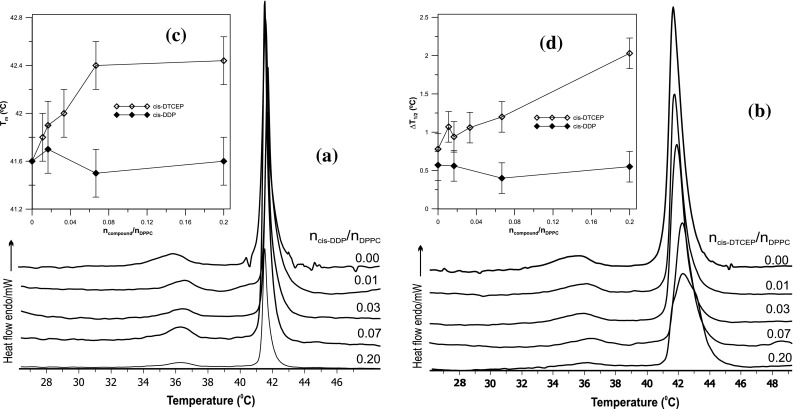
DSC transition curves of MLV with DPPC modified with the *cis*-DDP (**a**) and *cis*-DTCEP (**b**). Main phase transition temperature T_m_ (**c**) and half width ΔT_1/2_ peak (**d**) as a function of platinum(II) complexes concentration

The interaction of the antitumor drug cisplatin with phospholipid has been described for the first time by Speelmans (Speelmans et al. 1996) and further investigated by various authors in later years (Jensen and Nerdal 2008; Alves et al. 2016; Bourgaux and Couvreur 2014; Nierzwicki et al. 2015). Our DSC results for *cis*-DDP are in agreement with conclusions from the above mentioned earlier research. As can be seen in Fig. 2a, which shows DSC results for *cis*-DDP, this compound does not change the temperature of the main phase transition and only slightly increases its cooperativity. Platinum(II) complex with TCEP, similarly to *cis*-DDP, does not abolish the pretransition (see Fig. 2b); however the temperature of the main transition of DPPC is slightly increased whereas its cooperativity is decreased (Fig. 2c,d). This may indicate that *cis*-DTCEP, similarly to cisplatin, tends to remain in the polar head group region causing a decrease in flexibility of the bilayer although the new complex seems to penetrate the bilayer deeper. At the *cis*-DTCEP/DPPC molar ratio of 0.2 the main phase transition becomes wide and asymmetric probably due to formation of domains with a different amount of the complex.

To find out where exactly the *cis*-DTCEP locates in the bilayer and to explain its molecular interactions with DPPC the infrared spectroscopy was employed (Pentak 2014; Wu et al. 2011;

Cieślik-Boczula et al. 2012). Measurements were done in various temperatures between 25 and 50 °C to assess the influence of the investigated complex on the DPPC bilayer’s structure, both in gel and liquid crystal phase and to check how the compound’s presence impacts the main phase transition of liposomes (Pentak 2014).

**Fig. 3:**
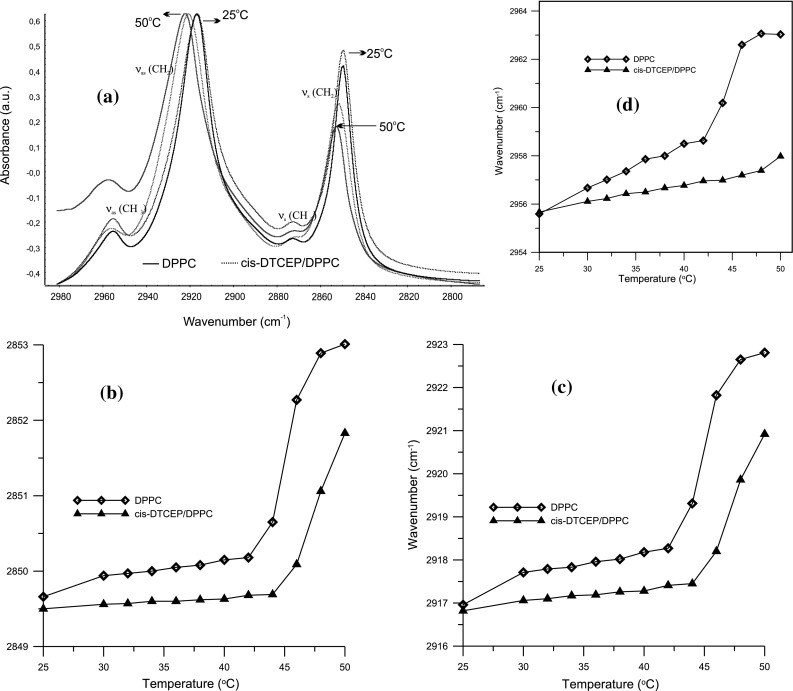
Infrared spectra of DPPC bilayer and DPPC with platinum(II) complex in the region between 3000 and 2800 cm^−1^ at the gel (25 °C) and liquid crystalline (50 °C) phases (**a**). Temperature dependence of the frequency of the asymmetric (**b**) and symmetric (**c**) CH_2_ and asymmetric CH_3_ (**d**) stretching modes in the presence and absence of *cis*-DTCEP for DPPC bilayers

The application of FTIR to the study of the phase behavior of DPPC membranes allows monitoring of various functional groups in order to obtain information about lipid-platinum(II) complex interactions at the molecular level. The most intense vibrational spectral region (3000–2900 cm^−1^) contains several stretching vibrations of C–H groups of phospholipid hydrocarbon chains. In the spectrum of DPPC liposomes the CH_2_ asymmetric stretching is located at about 2920 cm^−1^ whereas the symmetric one is located at about 2850 cm^−1^. Frequencies and widths of these bands are sensitive to conformational changes of lipid chains. This conformational fluidization of DPPC bilayer can be caused by the increase of temperature, water content, or by incorporation of compounds into the lipid membrane. The increase in the wavenumber of these bands testifies to increased liquidity of the hydrophobic part of the membrane. They respond to any difference occurred in the *trans/gauche* ratio in acyl chains. Conformational changes can also be detected by examining asymmetric CH_3_ at 2965 cm^−1^ and symmetric CH_3_ at 2872 cm^−1^ bands which are stretching modes of the terminal methyl group. Figure 3. shows comparison of infrared spectra of DPPC and DPPC doped with *cis*-DTCEP in the region of hydrophobic bilayer. Dependence of the asymmetric and symmetric CH_2_ stretching vibration in pure DPPC and *cis*-DTCEP/DPPC systems as a function of temperature is shown in Fig. 3a and b respectively. Values for both symmetric and asymmetric vibrations in the presence of platinum complex are a bit lower than for pure DPPC (Table 1). The main phase transition which is very sharp for the pure DPPC becomes broader and moves towards higher temperatures (Fig. 2b, c, d). This suggests that some of the chains remain in the *trans* conformation rather than *gouche*, which may be caused by their restricted mobility.

**Table 1:** Selected bands of IR spectra of cis-DTCEP/DPPC system and DPPC

Temperature	25 °C		36 °C		50 °C	
Frequency (cm^−1^)	DPPC	DPPC + *cis*-DTCEP	DPPC	DPPC + *cis*-DTCEP	DPPC	DPPC + *cis*-DTCEP
*δ*(CH_2_)	1467.7	1467.7	1467.6	1467.7	1467.9	1467.2
*ν*_s_(N–C)	925.7	926.1	–	927.3	925.7	925.8
*ν*_as_(N–C)	970.6	970.5	970.2	970.4	970.1	969.7
*ν*_as_(CCN)_1013_	1014.5	1014.3	1014.5	1014.8	1016.0	1012.5
*ν*_s_(COC)	1062.7	1067.7	1064.8	1064.3	1067.9	1064.7
*ν*_s_(PO_2_^−^)	1087.5	1087.5	1087.8	1087.5	1086.1	1086.2
*ν*_as_(COC)_sn_	1170.2	1170.3	1172.3	1170.8	1172.6	1172.7
			1178.2	1176.3	1181.9	1178.2
*ν*_as_(PO_2_^−^)	1222.1	1222.0	1222.7	1222.6	1222.7	1227. 9
					1226.7	1224.1
*ν*(C=O)	1738.6	1738.6	1738.9	1738.8	1739.1	1739.2
	1726.4	1726.4	1726.2	1726.1	1725.1	1726.5
*ν*_s_(CH_2_)	2849.7	2849.5	2850.1	2849.6	2852.9	2851.6
*ν*_s_(CH_3_)	2872.9	2872.9	2873.9	2873.3	2873.6	2872.9
*ν*_as_(CH_2_)	2917.0	2916.8	2917.9	2917.2	2922.7	2920.7
*ν*_as_(CH_3_)	2955.6	2955.7	2957.8	2956.5	2963.1	2957.4

*Vibrations* *δ* bending,* ν* stretching,* as* asymmetric,* s* symmetric

Moreover, conformational changes can also be detected by examining asymmetric and symmetric CH_3_ bands at 2956 and 2873 cm^−1^ respectively, which are stretching modes of the terminal methyl group. For pure DPPC ν(CH_3_) values increase with the change of the system’s phase. Example frequency values are shown in the Table 1, whereas, Fig. 3 illustrates how the frequency changes with the changing temperature. For pure DPPC we have ν_as_(CH_3_) = 2956.7 cm^−1^ for the gel phase and ν_as_(CH_3_) = 2963.1 cm^−1^ for the liquid crystal phase. After adding *cis*-DTCEP those frequencies change to ν_s_(CH_3_) = 2956.1 cm^−1^ and ν_s_(CH_3_) = 2957.4 cm^−1^ respectively, which signifies lower mobility of the chains in the presence of the compound. Additionally, the width of the vibrational bands was reduced after adding the compound which indicates that the bilayer liquidity was reduced as well. It is well known that the conformational disordering of an all-*trans* hydrocarbon chain is accompanied by both the upward shift in the ν_s_(CH_2_) and ν_as_(CH_2_) band maxima and the broadening of the overall band envelope. These changes reflect the increase in hydrocarbon chain conformational disorder and mobility that occurs with the onset of gauche rotamer formation and at the same time – decline in the number of all-*trans* rotamers (Jensen and Nerdal 2008).

The CH_2_ scissoring band is sensitive to the intermolecular forces and can serve a key band for examining the state of lateral packing of the methylene chains in various phases (Alves et al. 2016). As it is apparent from Table 1, δ(CH_2_) values do not differ significantly for pure and *cis*-DTCEP-doped DPPC which leads to the conclusion that the addition of the complex does not significantly change the structure of the DPPC model membrane. Probably the van der Waals interactions between DPPC alkyl chains *cis*-DTCEP are responsible for the membrane rigidifying effect. Thus, the IR studies confirmed the calorimetric investigations which registered a slight increase of temperature of the main phase transition of DPPC.

Spectral modes arising from the head group and interfacial region of lipid can also provide valuable information. The band of C=O stretching is very useful for probing the interfacial region, because ester groups are located between polar and non-polar interfaces in the structure of the DPPC.

**Fig. 4:**
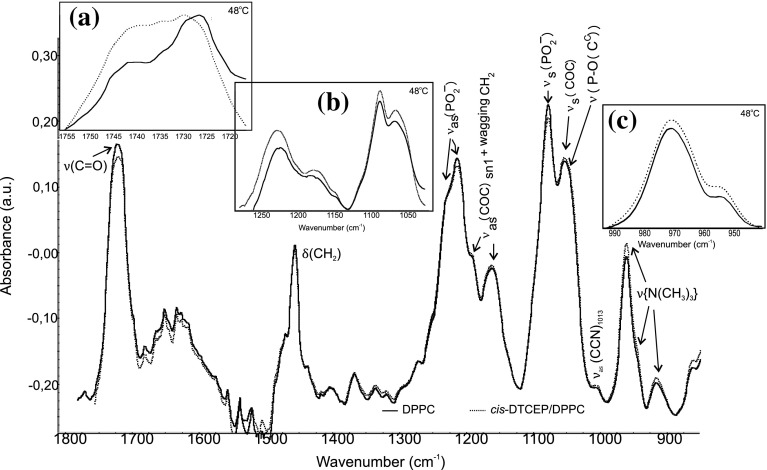
Infrared spectra of hydrated DPPC bilayer (*solid line*) and DPPC with *cis*-DTCEP (*dotted line*) in the region between 1800 and 800 cm^−1^ in the gel (25 °C) and liquid (48 °C) phases: C=O stretching (**a**), phosphate (**b**), and choline (**c**) bands

The most intense of these bands are the C=O stretching frequencies between 1750 and 1700 cm^−1^. The position of the ν(C=O) band’s maximum is sensitive to the conformation of ester groups and to the hydration level of the carbonyl region in the DPPC bilayer. Examining the C=O stretching band provides information about the strength of the hydrogen bonding (Lee and Chapman 1986; Lewis et al. 1994). The C=O stretching band for hydrated DPPC molecules forming the bilayer at gel phase consists of two subcomponent peaks (Lewis et al. 1994; 31]. The first subcomponent at a lower wavenumber may correspond to the hydrogen bounded carbonyl groups, while the high wavenumber band arises from no hydrogen groups (Binder 2003). The presence of *cis*-DTCEP in the DPPC lipid membrane below the main phase transition temperature induces only slight changes in the C=O stretching band (Table 1; Fig. 4). Above this temperature the frequency of the subcomponent corresponds to the hydrogen bond is lower for pure DPPC than for *cis*-DTCEP/DPPC system. This may indicate that there is a slight change of the hydration in the area of carbonyl groups of the lipid bilayer.

**Fig. 5:**
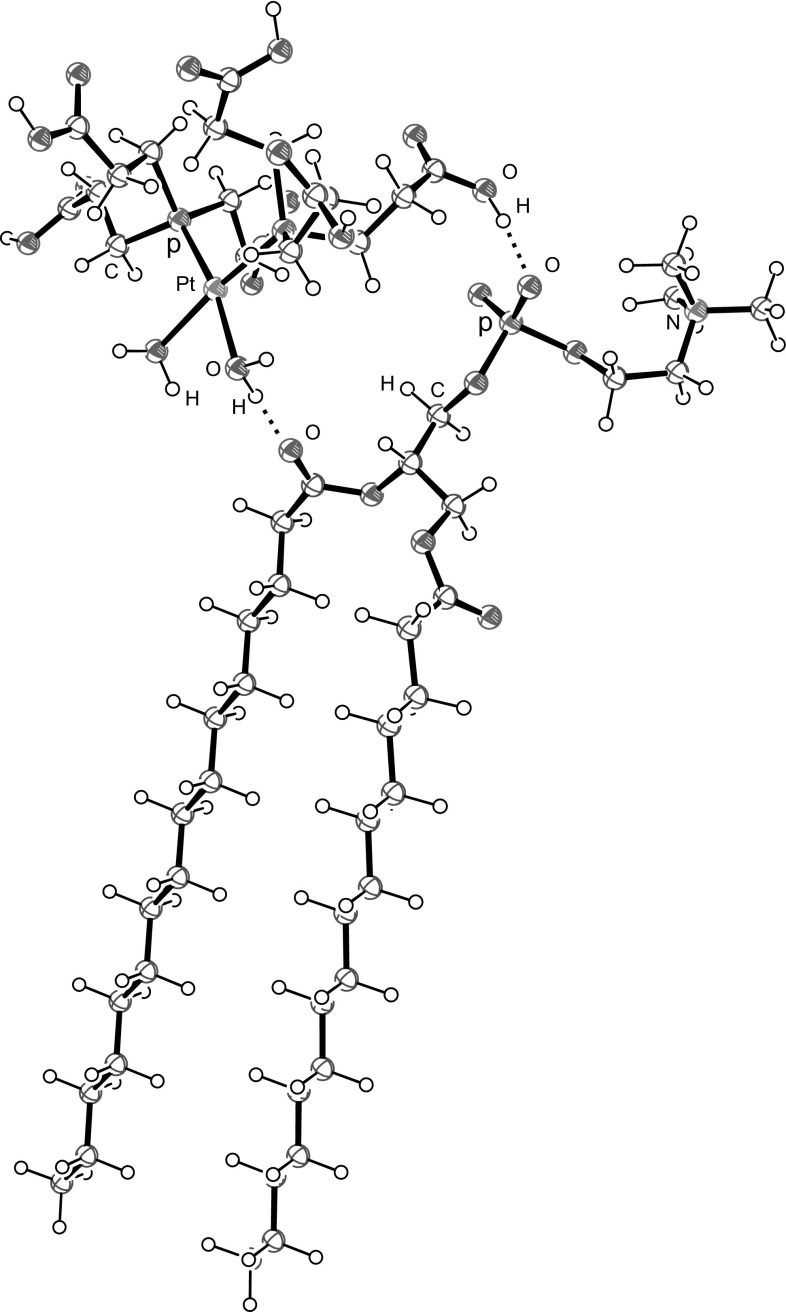
Formation of hydrogen bonds between DPPC and *cis*-[Pt(OH_2_)_2_{P(CH_2_CH_2_COOH)_3_}_2_]^2+^. Hydrogen atoms of CH_2_ groups of phosphine ligands have been omitted because of clarity

The interaction of *cis*-DTCEP and the head group of DPPC bilayer was monitored by analyzing the symmetric and asymmetric phosphate as well as choline stretching bands (Fig. 4). The frequency range from 1200 to 1260 cm^−1^ corresponds to the asymmetric stretching vibration of the PO_2_^−^ phosphate groups (Fig. 4). In the presence of *cis*-DTCEP there are no visible changes of frequencies for temperatures below the main phase transition whereas for the liquid phase the frequency of oscillation is slightly shifted to higher values (Table 1). Similarly low increase of the wave number was observed for ν_s_(PO_2_^−^). Additionally, in the presence of *cis*-DTCEP the band of the phosphate group is much broader than for pure DPPC indicating that there is an interaction between the investigated compound and lipid. The last observed band in the polar part of DPPC spectra was the band corresponding to the vibration of choline groups with maximum at 970 cm^−1^ for ν_as_(N–C). Analysis revealed that *cis*-DTEP induces very slight changes in this band and only in liquid phase (Table 1; Fig. 4). These results indicate that *cis*-[PtCl_2_{P(CH_2_CH_2_COOH)_3_}_2_] and ionic and neutral aqua complexes formed in the presence of water in the substitution reaction of chloride ligands by H_2_O molecules and dissociation of COOH groups: *cis*-[Pt(OH_2_)_2_{P(CH_2_CH_2_COOH)_3−n_(CH_2_CH_2_COO)_n_}_2_]^(2n−2)−^ interact with the head groups of DPPC molecules forming hydrogen bonds with COO groups of glycerol and PO_2_^−^ group (Fig. 5). There is also possible electrostatic interaction between negative ends of phosphine CH_2_CH_2_COO^−^ and the quaternary ammonium group. One molecule of platinum complex can interact with two heads of DPPC molecules leading to stabilization of DPPC bilayer structure as testified by increasing phase transition temperature.

Observed changes suggest that the investigated complex interacts with the polar group of the lipid bilayer and thus limits the mobility of the hydrocarbon tail. Studies of the interaction between cisplatin and model membranes, conducted for many years have demonstrated that *cis*-DDT forms stable complex with lipids due to electrostatic forces between the compound and head group of phospholipids (Jensen and Nerdal 2008; Alves et al. 2016). NMR study showed that *cis*-DDP preferentially interacts with the DMPC bilayer surface (accumulates on the bilayer surface), showing a low tendency to enter within the hydrophobic core of the bilayer (Bourgaux and Couvreur 2014). Besides, DSC measurements showed that *cis*-DDP increases stiffness of the membrane resulting in increased temperature of the main phase transition. The newly investigated compound—similarly to *cis*-DDP—interacts mainly with the DPPC head group not only by means of electrostatic forces. *Cis*-DTCEP bound with polar heads of lipids will also increase orderly hydrophobic chains in bilayer decreasing thereby the fluidity of the membrane which plays a crucial role in a correct distribution and function of some proteins. Therefore as a result of alteration in membrane fluidity functional properties of cells may be changed and apoptotic pathways that can result in cell death can be induced (Alves et al. 2016). Perhaps toxicity of the compound which we investigate can be attributed to the changes of the membrane fluidity however obtaining more precise information that would confirm this supposition requires application of a variety of membrane mimetic models, which is in our further plans.

## Conclusions

The objective of our work was to investigate molecular interactions between newly obtained platinum(II) with tris(2-carboxyethyl)phosphine complex (*cis*-DTCEP) and DNA and phospholipids as well as to check how they compare against diamminedichloroplatinum (II) (cisplatin), a well-known chemotherapeutic drug.

We started with fundamental research on a relatively simple complex-lipid and complex-DNA systems to be able to draw conclusions about the mechanisms of action of the *cis*-DTCEP complex, i.e., whether it works in the same way as cisplatin or not. These basic physicochemical tests were necessary before we could go further with our research and start investigating the effect of liposome-encapsulated platinum(II) complexes on cells.

The combined IR and DSC studies of *cis*-DTCEP/DPPC showed that, the *cis*-DDP tends to remain in the polar head group region causing a decrease in flexibility of the bilayer, the investigated new compound enters into the hydrophilic region of DPPC. *Cis*-DTCEP probably interacts with the head groups of DPPC molecules forming hydrogen bonds with COO groups of glycerol and PO_2_- group. One molecule of platinum complex can interact with two heads of DPPC molecules leading to stabilization of DPPC bilayer structure as testified by increasing phase transition temperature.

The binding and interactions of *cis*-DTCEP with plasmid DNA were investigated using TCSPC-FCS. The binding of DNA to the *cis*-DTCEP compound resulted in a stronger folding effect than for *cis*-DDP.

Obtained results illustrate that *cis*-DTCEP is a promising candidate for further evaluation, and may provide a novel therapeutic approach for the treatment of many cancer cells although further investigations are necessary to elucidate its cytotoxic activity.
